# Functional Analysis of PSRP1, the Chloroplast Homolog of a Cyanobacterial Ribosome Hibernation Factor

**DOI:** 10.3390/plants9020209

**Published:** 2020-02-06

**Authors:** Kevin Swift, Prakitchai Chotewutmontri, Susan Belcher, Rosalind Williams-Carrier, Alice Barkan

**Affiliations:** Institute of Molecular Biology, University of Oregon, Eugene, OR 97403, USA; kev.a.swift@gmail.com (K.S.); pchotewu@uoregon.edu (P.C.); sbelcher@uoregon.edu (S.B.); rozzz@uoregon.edu (R.W.-C.)

**Keywords:** chloroplast, ribosome, translation, plastid, light regulation

## Abstract

Bacterial ribosome hibernation factors sequester ribosomes in an inactive state during the stationary phase and in response to stress. The cyanobacterial ribosome hibernation factor LrtA has been suggested to inactivate ribosomes in the dark and to be important for post-stress survival. In this study, we addressed the hypothesis that Plastid Specific Ribosomal Protein 1 (PSRP1), the chloroplast-localized LrtA homolog in plants, contributes to the global repression of chloroplast translation that occurs when plants are shifted from light to dark. We found that the abundance of PSRP1 and its association with ribosomes were similar in the light and the dark. Maize mutants lacking PSRP1 were phenotypically normal under standard laboratory growth conditions. Furthermore, the absence of PSRP1 did not alter the distribution of chloroplast ribosomes among monosomes and polysomes in the light or in the dark, and did not affect the light-regulated synthesis of the chloroplast *psbA* gene product. These results suggest that PSRP1 does not play a significant role in the regulation of chloroplast translation by light. As such, the physiological driving force for the retention of PSRP1 during chloroplast evolution remains unclear.

## 1. Introduction

In photosynthetic eukaryotes, photosynthesis takes place inside chloroplasts, specialized organelles descended from cyanobacteria. Chloroplasts retain a highly reduced form of their ancestral bacterial genome, which encodes subunits of photosynthetic enzymes and the chloroplast gene expression machinery [1]. Many of the multimeric complexes involved in photosynthesis and chloroplast gene expression are of dual genetic origin, in that some subunits are encoded in the chloroplast genome and others in the nuclear genome. Thus, the biogenesis of the photosynthetic apparatus necessitates mechanisms to coordinate the activities of these physically separate genetic systems [2].

Plants rely on light to drive ATP synthesis and carbon assimilation, but light intensity and spectral quality are highly variable. Protein synthesis is an energy intensive process, so mechanisms have evolved to limit translation in plants when light is limiting. For example, the average size of cytosolic polysomes decreases shortly after shifting plants from light to dark [3,4], and translation in chloroplasts is globally repressed in the dark at the elongation and initiation steps [4]. The mechanisms underlying this global regulation of chloroplast translation in response to light are not known.

In this study, we address the hypothesis that Plastid Specific Ribosomal Protein 1 (PSRP1) globally represses chloroplast protein synthesis in the dark. PSRP1 is a nucleus-encoded protein that was originally found in a proteomic analysis of chloroplast 30S ribosomal subunits, when it was assumed to be a bone fide ribosomal protein [5]. Subsequently, it became clear that PSRP1 belongs to a family of proteins called ribosome hibernation factors, the best studied example of which is Ribosome Associated Inhibitor 1 (Rai1) or pY in *E. coli* [6,7,8]. During stationary phase and at low temperatures, Rai1 binds the 30S ribosomal subunit, which induces a conformational change that favors the stable association of 30S and 50S subunits into inactive 70S ribosomes (reviewed in 7,8). Despite the strong conservation of Rai1-related factors in bacteria, bacterial mutants lacking them show only subtle changes in phenotype and their physiological roles remain unclear. Proposed functions include global translational repression in response to stress, the protection of inactive ribosomes, and the rapid recovery of protein synthesis following stress-induced inhibition. The PSRP1 sequence places it in the “long Hibernation Promoting Factor” (l-HPF) subclass, members of which generally promote the formation of inactive 100S ribosome dimers [8]. Structural and biochemical data show, however, that PSRP1 binds and inactivates 70S ribosomes in a manner analogous to Rai1/pY [6,7,8,9,10,11]. PSRP1 binds the intersubunit space of chloroplast ribosomes, preventing access of tRNAs to the A and P sites [9,10]. However, the effects of PSRP1 on translation in vivo have not been reported. 

The PSRP1 homolog in cyanobacteria was named Light Repressed Transcript A (LrtA) because the abundance of its mRNA increases dramatically in the dark [12,13]. Deletion of *lrtA* in *Synechocystis* had no impact on growth rate under standard laboratory conditions, but slowed recovery after periods of starvation [13]. Expression of LrtA in *Synechococcus* (also known as HPF) is activated by the stringent response, a stress response pathway that facilitates adaptation to darkness in this organism [14]. *Synechococcus* LrtA promotes ribosome dimerization and a decrease in polysome content in the dark, but its effect on growth rate was not reported [14]. These observations led to the suggestion that PSRP1 represses chloroplast protein synthesis in the dark by maintaining 70S ribosomes in an inactive state [6,8,9]. In this study, we explored this possibility through biochemical and genetic analyses of PSRP1 in maize. 

## 2. Results and Discussion

### 2.1. Effects of Light-Dark Shifts on PSRP1 Abundance and Ribosome Association

**Figure 1:**
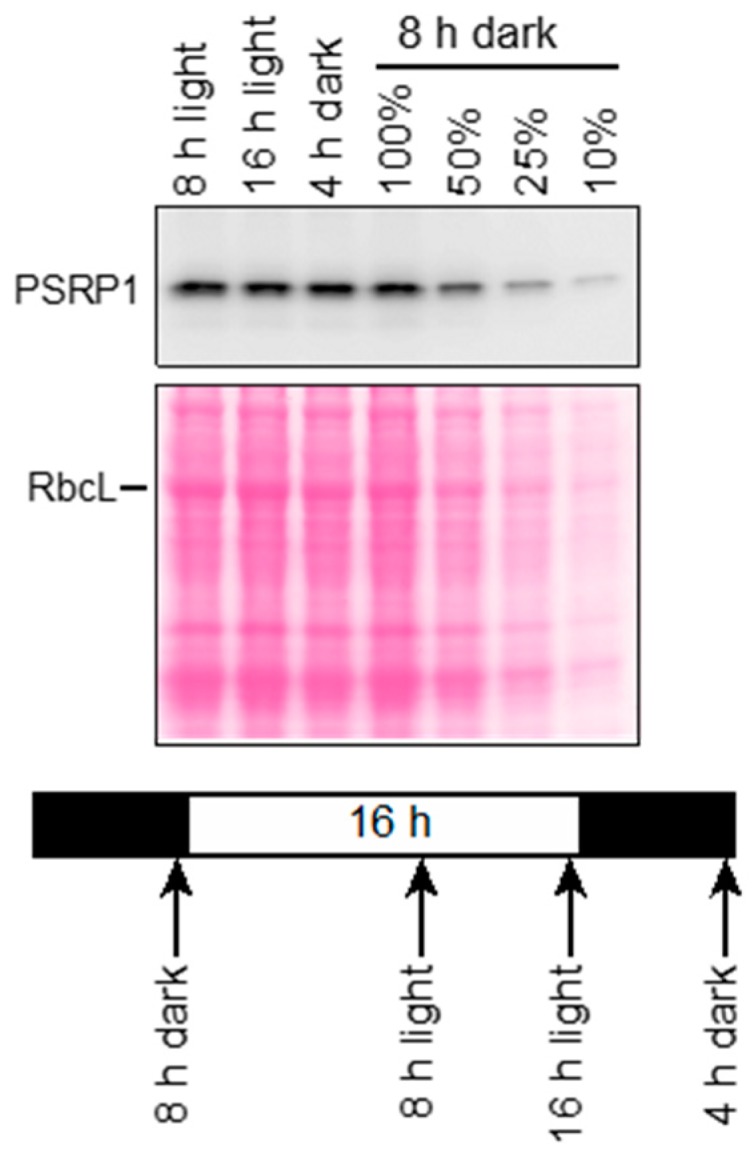
Plastid Specific Ribosomal Protein 1 (PSRP1) abundance during a diurnal cycle in maize. Seedlings (inbred line B73) were grown in diurnal cycles, and leaves were harvested at the indicated times on the eighth day after planting. Total leaf extracts were fractionated by SDS-PAGE, and PSRP1 was detected by immunoblotting. An image of the Ponceau S-stained filter is shown to illustrate equal sample loading and the abundance of the large subunit of Rubisco (RbcL).

The abundance of the *lrtA* mRNA in cyanobacteria decreases in the light [12,13]. By contrast, transcriptome data for the Arabidopsis gene encoding PSRP1 (AT5G24490) indicate that light has little impact on the abundance of PSRP1 mRNA (http://bar.utoronto.ca/efp/). To determine whether PSRP1 protein abundance changes in response to light, we used immunoblots to analyze PSRP1 levels in maize seedling leaves at two times during the day (8 and 16 h in the light) and two times during the night (4 and 8 h in the dark) (Figure 1). PSRP1 was found at similar levels in these samples, consistent with the Arabidopsis transcriptome data.

**Figure 2:**
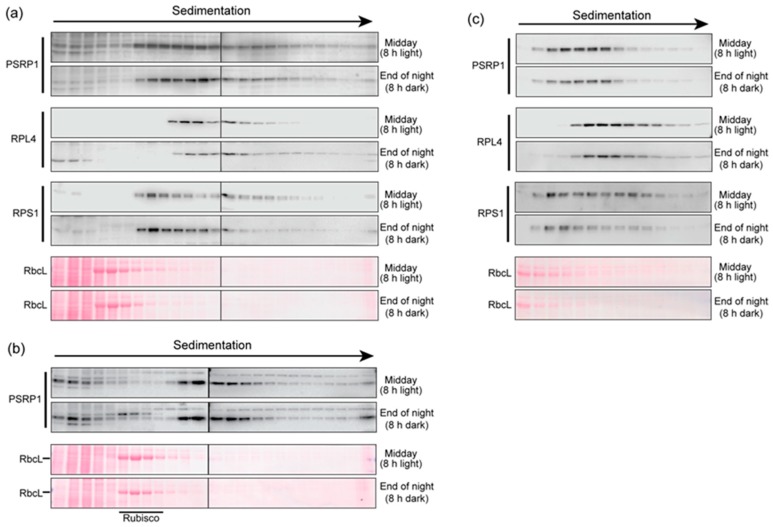
Sucrose gradient sedimentation of extracts of leaves harvested at midday (8 h light) or at the end of the night (8 h dark). The extraction buffer contained 10 mM Mg^++^ to maintain ribosome integrity, but lacked heparin and chloramphenicol, which are required to maintain polysome integrity (see text for rationale). Immunoblots of gradient fractions were probed to detect PSRP1, RPL4 (marker for the chloroplast 50S ribosomal subunit), or RPS1 (marker for the chloroplast 30S ribosomal subunit). In a revised nomenclature, chloroplast RPS1 and RPL4 are designated bS1c and uL4c, respectively [9]. Excerpts of the Ponceau S-stained blots are shown to illustrate the position of Rubisco in the gradients (illustrated by its RbcL subunit). (**a**) and (**b**) The complete set of fractions from two replicate experiments were divided between two gels (demarcated by a line). The gradient in panel (**a**) was centrifuged for 105 min, whereas the gradient in panel (**b**) was centrifuged for 150 min, resulting in deeper sedimentation of PSRP1 and marker proteins. (**c**) The peak PSRP1 fractions from a third experiment (fractions 6 through 19) are displayed on a single gel. The sedimentation conditions were the same as those used in panel (**a**).

We then addressed the possibility that the association of PSRP1 with ribosomes changes in response to light. Extracts of leaves harvested at midday or at the end of the night were fractionated by sedimentation through sucrose gradients under conditions that resolved monosomes and free ribosomal subunits in the middle of the gradient (Figure 2). Prior data show that PSRP1 binds 30S ribosomal subunits and 70S ribosomes, and suggest that it could not bind ribosomes in the process of translation [6,8,9,10,15]. Therefore, we did not aim to maintain polysome integrity in these assays. Whereas polysome extraction buffers typically include heparin to inhibit ribonucleases, we excluded heparin due to concern that it might weaken PSRP1-ribosome interactions. We also excluded chloramphenicol, which is included in polysome analyses to prevent ribosome runoff. Immunoblot analysis showed that marker proteins of the small and large chloroplast ribosomal subunits (RPS1 and RPL4, respectively) were found in overlapping peaks that sediment somewhat faster than Rubisco (~500 kDa), as expected for 30S, 50S, and 70S ribosomes (Figure 2a,c). The PSRP1 peak overlapped with both the RPS1 and RPL4 peaks. These results are consistent with the fact that PSRP1 binds both free 30S subunits and 70S monosomes [5,9,10]. PSRP1’s sedimentation behavior was similar in plants harvested at midday (8 h in the light) or before dawn (8 h in the dark). In replicate experiments (Figure 2a,b), only a small amount of unbound PSRP1 was observed near the top of the gradient. These results suggest that PSRP1’s association with ribosomes does not change in response to light.

### 2.2. Maize Mutants Lacking PSRP1 are Phenotypically Normal under Nonstress Growth Conditions

**Figure 3:**
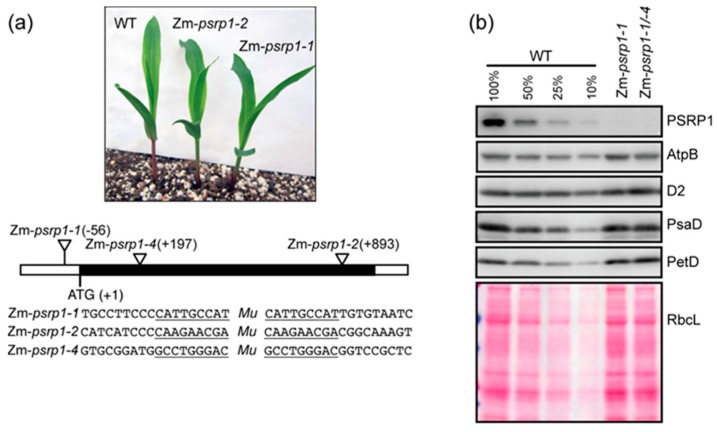
Maize *psrp1* mutants. (**a**) Mutant and wild-type (WT) (inbred line B73) seedlings at the developmental stage used for experiments reported here. The positions of the *Mu* insertions in each allele are shown below, with the target site duplications underlined. The black rectangle indicates the single exon in the gene. Additional images of mutant seedlings are shown in Figure S1. (**b**) Immunoblots showing the abundance of PSRP1 and representative subunits of photosynthetic complexes in Zm-*psrp1* mutants. Replicate blots were probed to detect AtpB, D2, PsaD, and PetD (subunits of the ATP synthase, Photosystem II, Photosystem I, and the cytochrome *b_6_f* complex, respectively). An image of one of the blots stained with Ponceau S is shown below to illustrate equal sample loading and the abundance of the large subunit of Rubisco (RbcL). An immunoblot demonstrating the absence of PSRP1 in Zm-*psrp1-2* homozygotes is shown in Figure 4a.

To further examine the functions of PSRP1, we analyzed transposon insertion alleles in the maize gene encoding PSRP1 (GRMZM2G347956 or Zm00001d034897, in B73 RefGen 3 or 4, respectively). The homozygous mutants and the heteroallelic progeny of a complementation test cross had no visible mutant phenotype at the seedling stage when grown under moderate light and temperature conditions (Figure 3a and Figure S1). The mutants grew to maturity in the field with no obvious phenotype, and set seed normally. Immunoblot analysis (Figure 3b) confirmed the absence of PSRP1 in these mutants. Therefore, similar to what has been observed for its bacterial relatives, PSRP1 has little impact on plant growth under nonstress growth conditions. Furthermore, immunoblot analysis showed that the accumulation of representative subunits of photosynthetic complexes harboring plastid-encoded subunits is similar in the mutants and wild-type (WT), suggesting that PSRP1 has little (if any) effect on chloroplast protein synthesis in seedlings grown under these controlled conditions.

### 2.3. Effects of PSRP1 on Chloroplast Translation in Light and Dark

**Figure 4:**
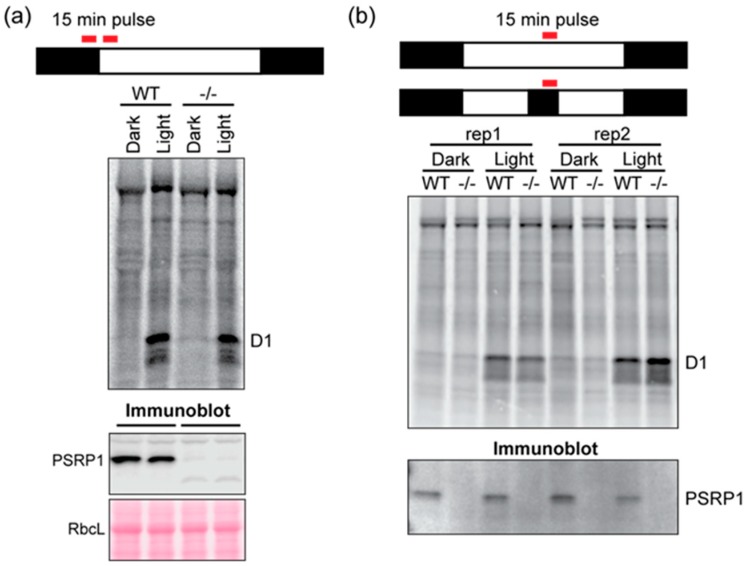
Pulse-labeling analysis of Zm-*psrp1-2* mutants in light and dark. Seedlings were pulse-labeled for 15 min at the end of the night (8 h dark) and after 15 min of illumination at dawn (**a**) or at midday (8 h in the light) or after one hour in the dark (**b**). Total leaf proteins were resolved by SDS-PAGE and transferred to nitrocellulose for phosphorimaging. The nitrocellulose was subsequently analyzed by immunoblotting (bottom panels) to validate the absence of PSRP1 in the mutant samples (-/-). WT siblings served as the control for the experiment performed at dawn, and the inbred line B73 served as the control for the experiment performed at midday.

Chloroplast translation initiation and elongation are globally repressed shortly after shifting plants from the light to the dark [4]. It has been hypothesized that PSRP1 contributes to this repression by maintaining 70S ribosomes in an inactive state [6,9]. To test whether PSRP1 is necessary for the repression of chloroplast translation in the dark, we compared protein synthesis in Zm-*psrp1* mutant and wild-type seedlings by pulse-labeling plants in the light and dark. In one experiment, we analyzed plants at the end of the night and after 15 min of illumination at dawn (Figure 4a). In a second experiment, we analyzed plants at midday in the light or after one hour in the dark (Figure 4b). The Photosystem II reaction center protein D1 is the most rapidly synthesized chloroplast protein in this light condition, and was the only chloroplast-encoded protein that could be unambiguously identified. The results show that D1 synthesis is repressed in the dark and induced in the light in a manner that is indistinguishable in the wild-type and mutant.

**Figure 5:**
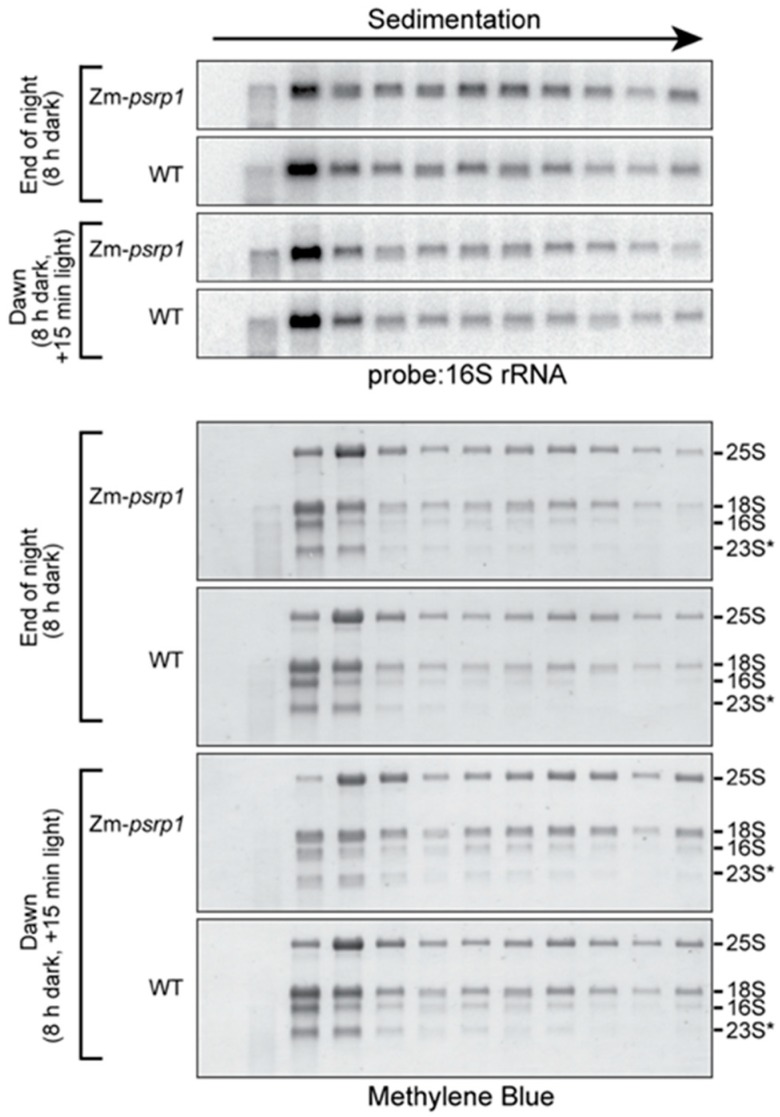
Sucrose gradient fractionation of polysomes in WT and Zm-*psrp1-2* seedling leaf extract at the end of night (8 h dark) and after 15 min of illumination at dawn. These experiments differed from those in Figure 2 by the inclusion of heparin and chloramphenicol to maintain polysome integrity, and by the use of centrifugation conditions that resolve polysomes rather than free ribosomal subunits. An equal proportion of the RNA recovered from each fraction was analyzed by RNA gel blot hybridization, using a probe specific for the chloroplast 16S rRNA (top). Images of the same blots stained with methylene blue are shown below to illustrate the distribution of all rRNAs in the gradient. 25S and 18S are cytosolic rRNAs, and 16S and 23S* are chloroplast rRNAs. The WT and mutant tissue came from siblings on the same ear.

The hypothesis that PSRP1 maintains 70S ribosomes in an inactive state predicts that the absence of PSRP1 would increase the proportion of ribosomes found in polysome fractions. To test this possibility, we fractionated total leaf extracts by sucrose gradient sedimentation under conditions that maintained polysome integrity. RNA was extracted from the gradient fractions and the 16S rRNA (a marker for chloroplast 30S ribosomal subunits) was detected by RNA gel blot hybridization (Figure 5). Lysates were prepared from seedlings harvested at the end of the night (8 h in the dark) and after 15 min of illumination at dawn. The sedimentation of chloroplast ribosomes (as marked by rRNAs) in the wild-type was similar in these two conditions. This is as expected, based on the prior demonstration of a plastome-wide decrease in translation elongation rate in the dark that maintained ribosome occupancy on all chloroplast mRNAs except *psbA* [4]. The mutant and wild-type data were also very similar under both conditions. These results strongly suggest that PSRP1 is not required to maintain 70S ribosomes in an inactive state in the dark. 

### 2.4. Conclusions

Photoautotrophs experience large fluctuations in energy supply during diurnal cycles. Protein synthesis in chloroplasts is globally inhibited in the dark [4], presumably as a means to conserve energy. PSRP1 emerged as a candidate factor that contributes to this global shut down of translation because it binds and inactivates 70S ribosomes, and its cyanobacterial homologs are induced in the dark and decrease the fraction of ribosomes found in polysomes [6,8,9,14]. However, our pulse-labeling analyses of maize *psrp1* mutants show that PSRP1 is not required to shut down chloroplast translation in the dark. Furthermore, the distribution of chloroplast ribosomes among monosome and polysome fractions was not detectably altered in the light or in the dark in *psrp1* mutants. In addition, we did not detect a difference in the association of PSRP1 with ribosomes at midday and at the end of the night, and PSRP1 abundance was similar during the day and night. 

There is evidence that bacterial ribosome hibernation factors are important for the efficient resuscitation of translation following stationary phase [7], so we considered the possibility that PSRP1 facilitates the rapid restoration of plastid translation after an extended period in the dark. For that purpose, we analyzed *psrp1* mutants by pulse-labeling and polysome analysis following the first 15 min of illumination in the morning. We did not detect any discernable difference between the mutant and wild-type under these conditions. Taken together, these results argue against the hypotheses that PSRP1 serves to repress chloroplast translation in the dark or to jump-start chloroplast translation after reillumination. 

The fact that PSRP1 has been retained in plant genomes since the divergence of chloroplasts and cyanobacteria implies that it plays an important role in plant physiology. PSRP1’s expression pattern may provide clues about its physiological role. The PSRP1 mRNA and protein are abundant in the seedling leaf tissues we assayed, but mRNA abundance is higher still in leaves of mature plants [16]. Transcriptome analyses also revealed a burst of expression in the maize seed shortly after pollination [17], and in young maize roots under drought-stress [18]. In a serendipitous observation made after completion of these experiments, a recently obtained Zm-*psrp1* allele in a different genetic background was slightly chlorotic, but only when grown without added fertilizer (Figure S1). This observation suggests a possible role for PSRP1 during nutrient stress. However, it remains possible that a mutation in a different gene accounts for this phenotype. Analysis of *psrp1* mutant phenotypes in a wide variety of tissues and developmental stages and in various stress conditions may reveal the *raison d’être* for this enigmatic ribosome hibernation factor.

## 3. Materials and Methods 

### 3.1. Plant Material

Maize PSRP1 is encoded by gene GRMZM2G347956 or Zm00001d034897 in B73 RefGen 3 and 4, respectively, and is orthologous to Arabidopsis AT5G24490 (see http://cas-pogs.uoregon.edu/#/pog/15142). The maize *psrp1* mutants were recovered during systematic sequencing of *Mu* insertion sites in the maize Photosynthetic Mutant Library, whose genetic background comes primarily from the inbred line B73 [19]. Plants were grown under 16-hour light (28 °C, ~250 µE) and 8-hour dark (26 °C) cycles, and leaf tissue was harvested when the third leaf was beginning to emerge (between seven and nine days after planting). Leaf tissue was flash frozen in liquid nitrogen and stored at -80 °C until use. The inbred line B73 was used for experiments that did not involve mutants. An additional insertion allele was obtained recently from the UniformMu collection (W22 genetic background), and is shown in Figure S1 to illustrate a possible background-dependent effect of nutrient stress on the mutant phenotype.

### 3.2. Immunoblotting, Antibodies, and Pulse-Labeling

Immunoblot analyses were performed as described previously [20]. Antibody to PSRP1 was raised to a recombinant protein fragment corresponding to amino acids 63-157 of the precursor form of maize PSRP1 (GRMZM2G347956_P01); this region was selected because it is highly conserved with Arabidopsis and lacks similarity to nonorthologous proteins. Polyclonal antibodies were raised in rabbits at Alpha Diagnostic International (San Antonio, Texas). The crude sera were affinity purified against the antigen using a Hitrap NHS-Activated affinity purification column (GE Healthcare, Little Chalfont). The antibodies to RPL4, RPS1, and D2 were obtained from Agrisera (catalog #AS15-3076, AS15-2875, and AS06-146, respectively). Antibodies to AtpB, PsaD, and PetD were generated by our group and have been described previously [21]. 

In vivo pulse-labeling of leaf proteins was performed as described in [4], using labeling periods of 15 min. Proteins were resolved in SDS-PAGE gels and transferred to nitrocellulose as for immunoblotting. The nitrocellulose was dried, and the radioactive proteins were detected with a phosphorimager. 

### 3.3. Sucrose Gradient and Polysome Analyses 

Polysomes were extracted and fractionated by sucrose gradient sedimentation as described previously [20]. RNA extracted from gradient fractions was analyzed by RNA gel blot hybridization, using a radiolabeled synthetic oligonucleotide complementary to the chloroplast 16S rRNA (positions 95249-95308 in the maize chloroplast genome). To analyze PSRP1’s association with ribosomes, the second and third seedling leaves were harvested at times indicated in figures, ground in liquid nitrogen, and thawed in buffer containing 25 mM HEPES-KOH pH 8, 60 mM KOAc, 10 mM MgOAc, 1% Triton X-100, 2 mM PMSF, 40 mM ß-mercaptoethanol, 2 μg/ml pepstatin, 2 μg/ml leupeptin, and 2 μg/ml aprotinin. The homogenate was filtered through glass wool to remove debris and centrifuged to pellet particulates. The supernatant was layered onto a 10–30% 4.4 ml sucrose gradient containing 18 mM HEPES-KOH pH 8, 10 mM MgOAc, and 180 mM KOAc, and centrifuged in a Beckman SW 55Ti rotor at 45,000 rpm for the times indicated in the legend to Figure 2. The gradients were fractionated into 24 samples and aliquots were assayed by immunoblot analysis. 

## Supplementary Materials

The following is available online at https://www.mdpi.com/2223-7747/9/2/209/s1, Figure S1: Images of maize *psrp1* mutants in different genetic backgrounds and grown under different conditions.

## References

[1] Bock R., Bock R. (2007). Structure, function, and inheritance of plastid genomes. Cell and Molec Biology of Plastids.

[2] Jarvis P., Lopez-Juez E. (2013). Biogenesis and homeostasis of chloroplasts and other plastids. Nat. Rev. Mol. Cell Biol..

[3] Juntawong P., Bailey-Serres J. (2012). Dynamic Light Regulation of Translation Status in Arabidopsis thaliana. Front. Plant. Sci.

[4] Chotewutmontri P., Barkan A. (2018). Multilevel effects of light on ribosome dynamics in chloroplasts program genome-wide and psbA-specific changes in translation. PLoS Genet..

[5] Yamaguchi K., von Knoblauch K., Subramanian A.R. (2000). The plastid ribosomal proteins. Identification of all the proteins in the 30 S subunit of an organelle ribosome (chloroplast). J. Biol. Chem..

[6] Sharma M.R., Dönhöfer A., Barat C., Marquez V., Datta P.P., Fucini P., Wilson D.N., Agrawal R.K. (2010). PSRP1 is not a ribosomal protein, but a ribosome-binding factor that is recycled by the ribosome-recycling factor (RRF) and elongation factor G (EF-G). J. Biol. Chem..

[7] Prossliner T., Skovbo Winther K., Sorensen M.A., Gerdes K. (2018). Ribosome Hibernation. Annu. Rev. Genet..

[8] Trösch R., Willmund F. (2019). The conserved theme of ribosome hibernation: From bacteria to chloroplasts of plants. Biol. Chem.

[9] Bieri P., Leibundgut M., Saurer M., Boehringer D., Ban N. (2017). The complete structure of the chloroplast 70S ribosome in complex with translation factor pY. EMBO J..

[10] Graf M., Arenz S., Huter P., Dönhöfer A., Novácek J., Wilson D.N. (2017). Cryo-EM structure of the spinach chloroplast ribosome reveals the location of plastid-specific ribosomal proteins and extensions. Nucleic Acids Res..

[11] Perez Boerema A., Aibara S., Paul B., Tobiasson V., Kimanius D., Forsberg B.O., Wallden K., Lindahl E., Amunts A. (2018). Structure of the chloroplast ribosome with chl-RRF and hibernation-promoting factor. Nat. Plants.

[12] Tan X., Varughese M., Widger W.R. (1994). A light-repressed transcript found in Synechococcus PCC 7002 is similar to a chloroplast-specific small subunit ribosomal protein and to a transcription modulator protein associated with sigma 54. J. Biol. Chem..

[13] Galmozzi C.V., Florencio F.J., Muro-Pastor M.I. (2016). The Cyanobacterial Ribosomal-Associated Protein LrtA Is Involved in Post-Stress Survival in Synechocystis sp. PCC 6803. PLoS ONE.

[14] Hood R.D., Higgins S.A., Flamholz A., Nichols R.J., Savage D.F. (2016). The stringent response regulates adaptation to darkness in the cyanobacterium Synechococcus elongatus. Proc. Natl. Acad. Sci. USA.

[15] Yamaguchi K., Subramanian A.R. (2000). The plastid ribosomal proteins. Identification of all the proteins in the 50 S subunit of an organelle ribosome (chloroplast). J. Biol. Chem..

[16] Downs G.S., Bi Y.M., Colasanti J., Wu W., Chen X., Zhu T., Rothstein S.J., Lukens L.N. (2013). A developmental transcriptional network for maize defines coexpression modules. Plant Physiol..

[17] Yi F., Gu W., Chen J., Song N., Gao X., Zhang X., Zhou Y., Ma X., Song W., Zhao H. (2019). High Temporal-Resolution Transcriptome Landscape of Early Maize Seed Development. Plant Cell.

[18] Opitz N., Paschold A., Marcon C., Malik W.A., Lanz C., Piepho H.P., Hochholdinger F. (2014). Transcriptomic complexity in young maize primary roots in response to low water potentials. BMC Genom..

[19] Belcher S., Williams-Carrier R., Stiffler N., Barkan A. (2015). Large-scale genetic analysis of chloroplast biogenesis in maize. Biochim. Biophys. Acta.

[20] Barkan A. (1998). Approaches to investigating nuclear genes that function in chloroplast biogenesis in land plants. Methods Enzymol..

[21] Pfalz J., Bayraktar O., Prikryl J., Barkan A. (2009). Site-specific binding of a PPR protein defines and stabilizes 5’ and 3’ mRNA termini in chloroplasts. EMBO J..

