# Supplementary material for: Functional Analysis of PSRP1, the Chloroplast Homolog of a Cyanobacterial Ribosome Hibernation Factor
Source: Plants (Basel). 2020 Feb 6;9(2):209. doi: 10.3390/plants9020209 (PMC7076655; doi:10.3390/plants9020209)

Figure S1. Images of maize *psrp1* mutants in different genetic backgrounds and grown under different conditions. The Zm-*psrp1-5* allele was obtained from the UniformMu (UFMu) resource (cluster: mu1069095, seed stock: UFMu-08577), which is in the W22 genetic background. The Zm-*psrp1-2* allele was used for experiments reported here, and is in a mixed genetic background that does not include W22. Plants in the pictured flats were grown for 20 days in the greenhouse with supplemental lighting (16 h days and 8 h nights). The night time low temperature was ~16 ˚C and the daytime high temperature was ~32 ˚C. Fertilizer was added to one set of plants 12 days after planting; the other set was maintained without fertilizer. The images show approximately twenty seedlings from ears of various genotypes and genetic backgrounds. The top photos show Zm-*psrp1-5* in comparison to the corresponding Uniform-Mu inbred line (W22 background) (left) and wild-type plants from a different genetic background (right). The *psrp1-5* mutants were pale green when not fertilized, but application of fertilizer at 12 days restored them to near-normal greenness. Importantly, it is possible that this phenotype results from a mutation in a gene other than *psrp1*, a possibility that will be addressed in the future with complementation test crosses. Photos on the bottom show the inbred line B73 and Zm-*psrp1-2* in two different mixed genetic backgrounds. These mutants are phenotypically normal in the presence or absence of fertilizer and vary in size according to genetic background.

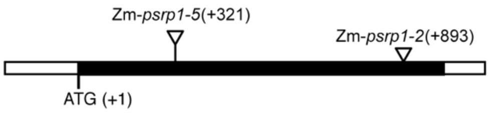

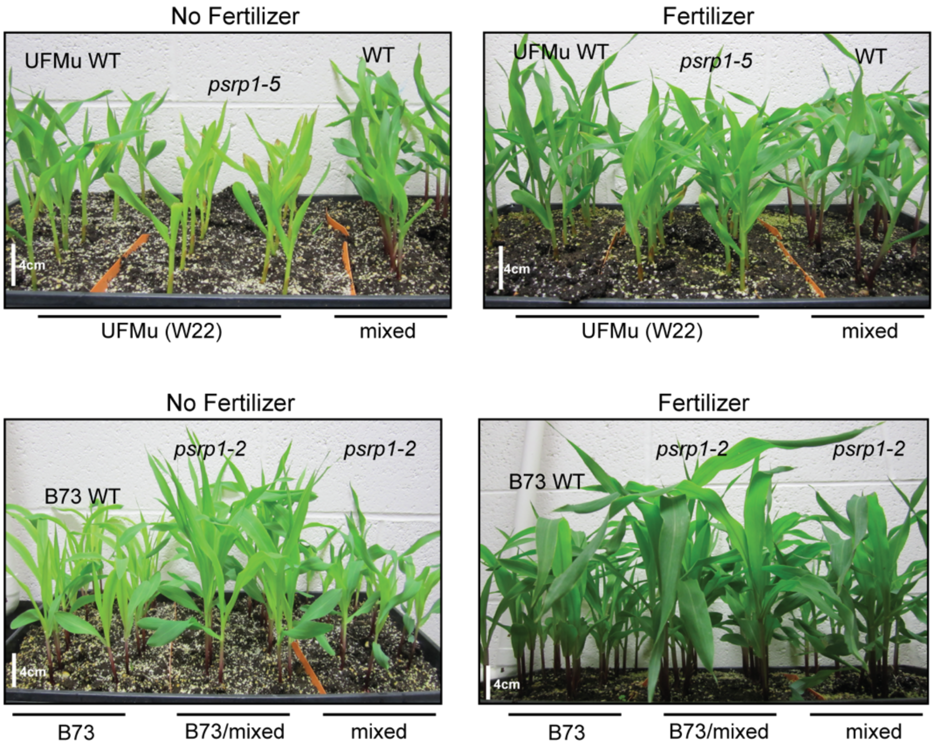

Supplement: Supplementary file 1 [file plants-09-00209-s001.pdf]
